# Summer scour syndrome in weaned dairy calves: case series

**DOI:** 10.1186/s13620-024-00273-0

**Published:** 2024-07-16

**Authors:** Rischi Robinson Male Here, Catherine McAloon, John Donlon, Mark McGee, Mary Duane, David Kenny, Bernadette Earley

**Affiliations:** 1 Animal & Grassland Research and Innovation Centre, Teagasc, Grange, Dunsany, Co. Meath, C15 PW93 Ireland; 2 School of Veterinary Medicine, University College Dublin, Dublin, Ireland

**Keywords:** Summer scour syndrome, Dairy calves, Blood ammonia, Inorganic nitrogen fertiliser

## Abstract

**Background:**

Summer scour syndrome (SSS) is a recently identified pathological condition affecting weaned dairy and dairy-beef calves during their first grazing season in Ireland. The syndrome is characterised by diarrhoea, weight loss, weakness, and can ultimately lead to death in some calves. Oral and oesophageal ulcerations are present in some cases. This study aimed to characterise a series of SSS cases in weaned dairy-bred calves on Irish commercial farms.

**Results:**

Five farms with calves having unexplained diarrhoea at grass were referred by private veterinary practitioners (PVP) following preliminary testing to exclude coccidiosis and parasitic gastroenteritis. Farms were visited within 2 to 5 days following PVP’s referrals, or 2 days to 3 weeks relative to the onset of clinical signs. Farm management data, grass and concentrate samples, and biological samples from 46 calves (8 to 10 calves/farm) displaying clinical signs were collected. Two farms were subsequently found positive for coccidiosis and/or had chronic pneumonia problems after a thorough herd investigation and were designated as non-case farms (NCF). The remaining three farms were deemed typical SSS outbreaks (case farms; CF). Mean rumen fluid pH per farm ranged from 6.67 to 7.09 on CF, and 6.43–6.88 on NCF. Mean rumen fluid ammonia concentrations ranged from 17.6 to 29.6 mg/L and 17.2–45.0 mg/L on CF and NCF, respectively. Corresponding blood ammonia concentrations ranged from 129 to 223 µmol/L and 22–25 µmol/L. Mean blood copper and molybdenum concentrations were within normal range on all farms. Grass crude protein concentrations on the paddocks where the calves had grazed, and were currently grazing on the day of visit ranged from 137 to 148 g/kg DM and 106–177 g/kg DM, respectively on CF, and 160–200 g/kg DM and 151–186 g/kg DM, respectively on NCF. On CF, inorganic nitrogen fertiliser was applied 1 to 3 weeks pre-grazing, whereas on the two NCF, inorganic nitrogen fertiliser was applied 2 to 3 weeks pre-grazing on one farm and no fertiliser was applied on the other.

**Conclusion:**

These findings suggest that copper or molybdenum toxicity, and ruminal acidosis are not the primary causes of SSS. High blood ammonia concentrations and the timing and level of inorganic nitrogen fertiliser application to paddocks pre-grazing, warrant further investigation.

**Supplementary Information:**

The online version contains supplementary material available at 10.1186/s13620-024-00273-0.

## Background

Summer scour syndrome (SSS) is a recently identified pathological condition that primarily affects both weaned dairy and dairy-beef calves during their first grazing season. Affected calves show clinical signs of weight loss, diarrhoea, weakness, oral and oesophageal ulcerations, and mortality are observed in some cases [1–3]. The syndrome is reported to occur in weaned calves within one-month post-turnout to grass or after moving to new pasture [3]. The term SSS is used widely in Ireland [1] and the United Kingdom (UK; [2, 3]) and very similar clinical presentations termed upper alimentary ulcerative syndrome (UAUS) have been documented in Australia [4]. In their surveillance report of 20 case submissions in the UK, Swinson et al. [3] found that while the overall prevalence of SSS was low, calf mortality in the groups of calves from which the cases were submitted varied from 2 to 40% and morbidity from 10 to 100%, and no consistent pathogens were identified in SSS calves. In Ireland and the UK [3], the syndrome is diagnosed after the common causes of calf diarrhoea at grass, including coccidiosis, parasitic gastroenteritis (PGE), infectious agents (e.g., bovine viral diarrhoea virus (BVDV), *Salmonella*), and copper or molybdenum toxicities, are excluded. In Australia, UAUS diagnosis is based on the presence of oesophageal necrosis lesions, with BVDV infection having been excluded [4]. The syndrome has not been reported in suckler cow-calf operations and appears to only occur in dairy or dairy-beef calves. The available literature to date suggests that the occurrence of the syndrome is similar across countries, with grass as a common exposure and artificially reared calves within their first grazing season. However, the primary cause/s of the syndrome remains unclear [1, 3, 4] and there is limited information available regarding the characteristics of the affected calves. The objectives of the present study were to characterise SSS in weaned dairy-bred calves and to determine the common conditions under which SSS occurs naturally on Irish commercial farms.

## Methods

Ethical approval for the present study was reviewed and granted by the Teagasc Animal Ethics Committee (TAEC0323-375).

### Case definition and veterinary referral

In May 2023, private veterinary practitioners (PVP) across Ireland were contacted and asked to refer potential SSS cases for investigation to the herd health section in the School of Veterinary Medicine, University College Dublin. Farms with potential SSS cases were defined as “*any dairy or dairy-beef farm with weaned calves displaying signs of unexplained diarrhoea at grass, and negative for coccidiosis or PGE*”. The case definition was developed in order to include farms with conditions that were typical of SSS. In the experience of the author (CMcA) investigating cases of calf diarrhoea at grass, SSS is usually a diagnosis that can only be given after other common plausible causes of diarrhoea in weaned calves at grass are ruled out. In June 2023, five farms were referred by the PVP’s with suspected SSS cases. History of the suspected SSS cases on each farm is summarised in Table 1. The farms were located in Counties Louth (Farms 1 and 4), Kilkenny (Farms 2 and 3), and Offaly (Farm 5).

**Table 1:** Overview of suspected SSS case history on each farm

History	Farm 1	Farm 2	Farm 3	Farm 4	Farm 5
Clinical signs	Lethargy and diarrhoea	Dullness and diarrhoea	Diarrhoea	Diarrhoea	Diarrhoea and ill-thrift
Onset of clinical signs^a^	ca. 6 weeks	ca. 4 weeks	ca. 6 weeks	ca. 2 weeks	ca. 2 weeks
Number of calves^b^	52	38	38	28	21
Affected calves moved indoors	Yes, four worst-affected calves	Yes, four worst-affected calves	Yes, eight worst-affected calves	Yes, seven worst-affected calves	Yes^e^
Treatments	Increased concentrate feeding (from 1 to 1.5 kg/h/d); calcium carbonate buffer and coccidiostat^d^	Oral yeast supplement (Ruminate powders; Acravet, Ireland)	Re-introduced hay at grass	Oral sulfadimidine antibiotic and yeast supplement (Ruminate powders; Acravet, Ireland)	Coccidiostat and anthelmintic
Moved paddock^c^	Yes	Yes	No	No	Yes
Farm health history	Cryptosporidiosis in February 2023 and bovine respiratory disease (BRD) in April 2023 before turnout	SSS type of clinical signs observed over the past 3 years	SSS type clinical signs observed over the past 4 years	SSS type clinical signs observed in the previous year (2022)	Lungworm health problems in the previous year (2022)

ca. – circa; ^a^ Observed by the farmers, relative to turnout; ^b^ Within the affected group; ^c^ Shortly after the onset of clinical signs; ^d^ Despite of negative for coccidiosis; ^e^ Number of affected calves that were moved indoor was not provided by the farmer

### Farm visits and sample collections

Farm visits were conducted within 2 to 5 days following the PVP’s referrals, or 2 days to 3 weeks since the farmers observed the onset of clinical signs within the group of affected calves. On the day of the farm visit, calves were grazing on their respective paddocks and had received concentrate supplementation earlier in the morning, before being brought indoors for clinical examinations and sample collection, as described below. The investigation protocol and samples collected from affected calves were the same across all farms. With the consent of the farmers, data relating to calf breed, date of birth, age, sex, and farm enterprise were retrieved from the Irish Cattle Breeding Federation (ICBF) database (www.icbf.com). An interview, by means of a questionnaire, was conducted with each farmer during the farm visits to record on-farm management practices, including calf nutrition, weaning procedures, fertiliser use, turnout to pasture and grazing management.

### Clinical examinations

During the farm visits, a general clinical examination was performed by a certified veterinarian (CMcA) on 8 to 10 affected calves per farm resulting in a total of 46 calves. The examined calves were randomly selected from calves with observed clinical signs in the affected group. Clinical variables (calf’s demeanor, mucous membranes, enophthalmos score, presence of nasal or eye discharge, respiratory and heart rate, hydration status, rumen contraction and filling, and rectal temperature) were assessed by visual observation, auscultation, and/or palpation. Calf body weight was estimated using a weigh band (Kamer, Ukal, Niederbronn-les-Bains, France). A focused thoracic ultrasound (Tecnoscan SR-1 C, Imporvet, Spain) was performed on the right thoracic side of each calf, and calves with area of lung consolidation  > 1 cm^2^ were considered to have pneumonia [5, 6].

### Faecal samples

Rectal faecal samples were collected, and the faecal consistency was assigned a score, as follows: (1) runny, liquid consistency, splatters on impact, spreads rapidly; (2) loose (watery), may pile slightly, spreads and splatters moderately on impact and settling; (3) soft, piles but spreads slightly on impact and settling; (4) dry, hard, dry appearance, original form not distorted on impact and settling. Calves with a faecal score of 1 or 2 were considered to have diarrhoea [7]. Faecal samples were pooled (3 to 5 samples from the same farm per pool) and analysed using the McMaster technique [8]. Farms with at least one faecal pool detected to have > 200 eggs/gram of faeces [8] or ≥ 500 oocysts/gram of faeces [9] were considered to have a high parasitic burden.

### Blood samples

Blood samples were collected via jugular venepuncture into vacutainer tubes: 1 × 4 ml K_3_EDTA, 3 × 9 ml Li Heparin (Greiner Vacuette; Cruinn Diagnostics, Dublin, Ireland), and 1 × 8.5 ml no-anticoagulant (BD SST II vacutainers; Unitech, Dublin, Ireland) and placed in a cool box (8^0^C). Immediately, following blood collection, 20 µL of whole blood from the K_3_EDTA tube was analysed for ammonia using a point-of-care analyser (PocketChem BA, Arkray, Japan), as described by Gultekin et al. [10] and Goggs et al. [11]. Whole blood K_3_EDTA samples were analysed for complete blood profiles using an ADVIA haematology analyser (ADVIA 2120, Siemens Healthineers, UK) as described by McGettigan et al. [12]. Plasma and serum samples were harvested from the Li Heparin and no-anticoagulant blood tubes, respectively, after centrifugation (1900 × g for 15 min at 4 °C) and stored at − 20 °C until analysis. Plasma samples were analysed for blood biochemical concentrations (total protein, creatinine kinase (CK), albumin, and globulin) using an automated chemistry analyser (Olympus AU480, Beckman Coulter Ltd., Co. Clare, Ireland), and blood urea nitrogen (BUN), gamma glutamyl transferase (GGT) and glutamate dehydrogenase (GLDH), using a clinical chemistry analyser (RX Imola, Randox Laboratories, UK). Serum samples were analysed for blood mineral profiles using Agilent 7850 analyser (FarmLab Diagnostic, Emlagh, Elphin, Co. Roscommon).

### Rumen fluid

Rumen fluid samples were collected using a modified trans-oesophageal tube, as described by Smith et al. [13]. Rumen pH was measured immediately using a digital pH meter (Orion Star A221; Thermo Fisher Scientific, United States) that has been calibrated at the start of each visit. Rumen fluid samples were preserved by transferring 4 ml of each sample to tubes containing 1 ml of 50% trichloroacetic acid (TCA) as described by Smith et al. [13]. Rumen ammonia, lactic acid, and volatile fatty acids (VFAs) concentrations were measured according to O’Connor et al. [14].

### Grass and concentrate samples

Herbage mass (kg dry matter (DM)/ha) on paddocks where calves had previously grazed (previous) and on paddocks where calves were grazing on the day of the farm visit (current) was estimated based on the sward height that was measured using a rising plate meter (Grasstec Ltd, Co. Cork, Ireland), as described by O’Connor et al. [14]. Representative samples of grass were collected from each paddock using an electric grass shears (Bosch Isio, Grasstec Ltd., Cork, Ireland) and placed in ziplock plastic bags before being transported to Teagasc, Grange. A sub-sample (∼ 200 g) of fresh grass was dried at 90^0^C for 24 h to determine the DM concentration, and another sub-sample (∼ 200 g) was dried at 40^0^C for 72 h and ground through a Retsch SM100 mill (1 mm aperture; Retsch GmbH, Haan, Germany) to determine chemical composition. Crude protein (CP), neutral detergent fibre (NDF), acid detergent fibre (ADF), ash and water-soluble carbohydrate (WSC) concentrations, and DM (DMD) and organic matter (OMD) digestibility was measured using wet chemistry methods [14]. Grass mineral composition was analysed using acid extraction method on Perkin Elmer NexION 2000 ICP-MS (FBA Laboratories Ltd., Co. Waterford, Ireland).

Concentrate samples (∼ 200 g) were collected in a ziplock plastic bag and transported to Teagasc, Grange. The samples were dried at 40^0^C for 72 h, ground through a CT 293 Cyclotec mill (1 mm aperture; FOSS, Denmark) and analysed for DM, CP, NDF, ash, and starch concentrations as described by O’Kiely [15].

### Statistical analysis

The data were analysed descriptively (PROC FREQ and PROC MEANS) using SAS 9.4 (SAS Institute Inc., Cary, North Carolina, United States).

## Results

Calves sampled on Farms 1, 2, and 4 had undetected or low parasitic burden, and satisfied the SSS case definition, and therefore were designated as case farms (CF; CF 1, CF 2, and CF 3, respectively). Calves sampled on Farm 3 had a high coccidial burden, and 50% were diagnosed with chronic pneumonia. Similarly on Farm 5, 63% of calves were diagnosed with chronic pneumonia, and the farm had evidence of mineral issues based on very high forage molybdenum. Consequently, Farm 3 and Farm 5 were designated as non-SSS case farms (NCF; NCF 1 and NCF 2, respectively).

### Farm managements

All CF were dairy farms with calves born during the spring calving season, whereas NCF 1 was a mixed enterprise farm (dairy cow and dairy-beef systems) and NCF 2 was a dairy-beef farm. All farms had BVDV-negative status, with calves tested for BVDV at birth as part of the national eradication programme. The summary of management practices and weaning procedures implemented on each farm are shown in Table 2.

**Table 2:** Farm description, management, and weaning procedures on CF and NCF

**Description/management**	**CF**			NCF	
	CF 1	CF 2	CF 3	NCF 1	NCF 2
Farm type	Dairy	Dairy	Dairy	Mixed dairy and dairy-beef	Dairy-beef
Calf breed	HO × FR	HO × FR	HO × FR	HO × FR; AA × HO, LM × HO; BA × HO	AA × FR
Calf source	Homebred	Homebred	Homebred	Homebred	Purchased (at 3 weeks of age)
Dam diarrhoea vaccination	Yes	Yes	Yes	Yes	N/A
Calves vaccination status	BRD	BRD	BRD	BRD	Clostridia
Coccidiostat^a^	Yes	Yes	Yes	Yes	Yes
Anthelmintic^a^	Yes	Yes	Yes	Yes	Yes
Water pre-weaning	Yes, ad libitum	Yes, ad libitum	Yes, ad libitum	Yes, ad libitum	Yes, ad libitum
Age water offered	1 day	7 days	7 days	1 day	At arrival (3 weeks)
Concentrate pre-weaning	Yes	Yes	Yes	Yes	Yes
Age concentrate offered	N/A	N/A	2 weeks	2 weeks	At arrival (3 weeks)
Forage pre-weaning	Yes, straw	Yes, straw	Yes, straw	Yes, hay	Yes, straw and silage
Method of milk feeding	Automatic feeder	Automatic feeder	Manual	Manual	Manual
Type of milk feeding	Milk replacer	Milk replacer	Whole milk	Transition milk; Milk replacer	Milk replacer
Amount of milk feeding	Gradually reduced milk feeding (amount at the start phase N/A)	3 L TAD at the start phase, and gradually reduced over the last 10 d to wean	5 L divided into two feedings for 4 weeks, then 5 L OAD and gradually reduced to 3 L and 1 L OAD over the last 10 d to wean	2 L TAD for 1 week (transition milk), then 3 L TAD (milk replacer)	3 L TAD, and then 3 L OAD over the last 7–10 d to wean
Decision for weaning	Age	Age	Age	Age	Age
Target weaning age on farm	10–12 weeks	10–12 weeks	10–12 weeks	10 weeks	11–12 weeks
Weaning procedure	Gradual^b^	Gradual^b^	Gradual^b^	Gradual^b^	Gradual^b^
Indoor weaning	Yes	Yes	Yes	Weaning at grass^c^	Yes
Immediate turnout post-weaning	Yes	No, stay indoor for 3 weeks	No, stay indoor for 2 weeks	Weaning at grass^c^	No, stay indoor for 1 week
Mineral supplementation at turnout	No	Yes	No	No	No
Concentrate at grass (amount)	Yes (1 kg/h/d)	Yes (1.5 kg/h/d)	Yes (1–2 kg/h/d)	Yes (1 kg/h/d)	Yes (2 kg/h/d)
Forage at grass	No	Yes, straw	No	Yes, straw	Yes, hay

HO – Holstein, FR – Friesian, LM – Limousin, AA – Aberdeen Angus, BA - Blonde D'Aquitane; BRD – bovine respiratory disease; N/A – not available; OAD – once a day; TAD – twice a day; ^a^ Timing of dosing was not provided by the farmers; ^b^ Step-down milk feeding; ^c^ Milk feeding continued at grass before fully weaned

On CF and NCF 1, inorganic nitrogen fertiliser was applied 1 to 3 weeks prior to calves grazing the paddocks (Table 3); however, the farmers did not have the record of fertiliser application rate. During the visits, granules of fertiliser were observed in some parts of the paddocks on CF 2. On NCF 2, no fertiliser was applied to the paddocks pre-grazing.

**Table 3:** Turnout date, grazing system, fertiliser applications, and herbage mass on CF and NCF

Description/management	CF			NCF	
	CF 1	CF 2	CF 3	NCF 1	NCF 2
Date of visit	14/06/2023	15/06/2023	23/06/2023	22/06/2023	27/06/2023
Turnout	15/04/2023	11/05/2023	25/05/2023	21/04/2023	15/05/2023
Duration of clinical signs^a^	ca. 2 weeks	2 days	ca. 2.5 weeks	ca. 2 weeks	ca. 3 weeks
Grazing system	Rotational grazing	Strip grazing	Rotational grazing	Rotational grazing	Strip grazing
Type of fertiliser applied^b^	Protected urea	NPK	Protected urea	NPK	No fertiliser
Timing of fertiliser application^b^	3 weeks	2 weeks	1 week	2–3 weeks	No fertiliser
Herbage mass (kg DM/ha)^c^	2629	423	1838	836	742

ca. – circa; NPK – nitrogen, phosphorus, and potassium; Rotational grazing – pasture was divided into several paddocks and grazing was rotated sequentially; Strip grazing – grazing was allocated per strip of the paddock using a movable electric fence; ^a^ Since the onset of clinical signs to farm visits; ^b^ On the paddocks where the onset of clinical signs were observed, prior to calves grazing the paddocks; ^c^ Herbage mass on paddocks where calves were grazing on the day of farm visit

### Grass and concentrates analyses

The grass and concentrate chemical composition, and grass trace minerals are presented in Table 4. Grass DM concentrations varied between farms, with grass samples from CF having higher DM concentrations compared to NCF. The CP concentrations in all grass samples were equal to or less than 200 g/kg DM, with the lowest CP in grass samples from the current paddock on CF 1. Grass samples from all farms had NDF concentrations above 400 g/kg DM, except for the previous and current paddocks on CF 2 and NCF 2, respectively. Grass WSC concentrations from the current and previous paddocks were numerically higher on CF compared to NCF. Grass molybdenum concentrations from NCF 1 (previous paddock) and NCF 2 exceeded the normal range. Grass copper concentrations were within the normal range on all farms except for grass samples from CF 1 and CF 3. Conversely, grass cobalt concentrations from most farms were above the normal range. The CP concentration of the concentrate supplements were similar between CF and NCF. Concentrates from CF 1 and NCF 1 had the highest NDF but the lowest starch content compared to the other farms.

**Table 4:** Grass and concentrate chemical composition, and grass trace minerals on CF and NCF

Analyses	CF					NCF				Normal references^c^
	CF 1		CF 2		CF 3	NCF 1		NCF 2		
	Current	Previous^a^	Current	Previous^a^	Current^a, b^	Current	Previous^a^	Current	Previous^a^	
***Grass chemical composition***										
DM (g/kg)^d^	268	308	272	254	245	258	244	179	187	–
CP (g/kg DM)	106	148	177	137	125	151	160	186	200	–
Ash (g/kg DM)	70	97	38	161	43	96	86	106	129	–
NDF (g/kg DM)	471	480	433	396	454	477	506	370	457	–
ADF (g/kg DM)	258	265	239	223	265	262	277	240	261	–
WSC (g/kg DM)	216	141	173	140	174	124	83	138	63	–
DMD (g/kg DM)	746	720	732	724	724	676	621	778	668	–
OMD (g/kg)	739	713	750	689	725	664	626	764	653	–
DOMD (g/kg DM)	688	643	723	565	692	596	597	679	564	–
***Grass mineral composition***										
Phosphorus (%)	0.24	0.34	0.33	0.33	0.22	0.25	0.31	0.43	0.35	0.25–0.50
Potassium (%)	2.20	2.28	2.74	2.85	1.57	2.17	2.11	2.65	2.64	1.7–3.3
Calcium (%)	0.45	0.78	0.65	0.55	0.15	0.53	0.60	1.01	0.76	0.38–0.75
Magnesium (%)	0.13	0.25	0.21	0.20	0.12	0.17	0.19	0.18	0.21	0.14–0.25
Sodium (%)	0.13	0.14	0.25	0.27	0.14	0.09	0.07	0.17	0.04	0.17–0.32
Sulphur (%)	0.20	0.30	0.34	0.35	0.20	0.21	0.26	0.32	0.32	0.18–0.35
Manganese (mg/kg)	106	137	71	117	68	174	136	74	64	100–200
Copper (mg/kg)	3.0	5.3	6.6	9.0	4.0	7.4	9.5	8.2	8.3	6–12
Zinc (mg/kg)	18	25	27	34	18	23	29	25	26	25–50
Iron (mg/kg)	183	721	916	523	285	599	312	105	633	120–300
Molybdenum (mg/kg)	1.5	2.3	1.8	1.0	1.0	2.2	3.2	5.1	11.9	1.5–2.6
Selenium (µg/kg)	65	78	95	96	33	59	101	158	142	70–140
Iodine (µg/kg)	305	537	396	561	347	458	669	205	306	200–420
Cobalt (µg/kg)	118	394	285	323	132	244	198	101	306	100–180
***Concentrate chemical composition***										
DM (g/kg)	884		861		886	825		866		
CP (g/kg DM)	173		184		193	192		177		–
Ash (g/kg DM)	102		70		74	87		64		–
NDF (g/kg DM)	260		188		164	239		183		–
Starch (g/kg DM)	147		320		317	180		274		–

DM – dry matter; CP – crude protein; NDF – neutral detergent fibre; ADF – acid detergent fibre; WSC – water soluble carbohydrate; DMD – dry matter digestibility; OMD – organic matter digestibility; DOMD – dry organic matter digestibility; ^a^ Paddocks where the onset of clinical signs were observed; ^b^ First paddock since turnout; ^c^ Reference values for grass mineral were sourced from FBA laboratories; ^d^ DM concentration after drying

### Clinical examinations

Calf characteristics and clinical data are shown in Table 5. Calves on CF predominantly had diarrhoea, weight loss, poor coat, and some calves had hyper-salivation. Traces of healed mouth ulcers were observed in four calves and one calf on CF 1 and CF 2, respectively. On NCF 1 and NCF 2, calves had clinical signs of diarrhoea and pneumonia.

**Table 5:** Calf characteristics and clinical data of calves on CF and NCF

Characteristics/clinical variables	CF			NCF	
	CF 1 (*n* = 10)	CF 2 (*n* = 10)	CF 3 (*n* = 10)	NCF 1 (*n* = 8)	NCF 2 (*n* = 8)
***Calf characteristics***					
Calf sex (number)	Female (10)	Female (10)	Female (10)	Female (3); Male (5)	Male (8)
Age at turnout to grass (day)	67 (6)	100 (4)	100 (4)	51 (13)	85 (11)
Age at visit (day)^a^	127 (6)	135 (4)	129 (4)	113 (13)	128 (11)
Body weight (kg)^b^	116 (12.4)	123 (11.7)	129 (10.2)	112 (10.8)	103 (21.4)
***Clinical examinations***					
Rectal body temperature (^0^C)	39.0 (0.2)	39.1 (0.4)	38.1 (1.0)	39.2 (1.0)	39.1 (0.3)
Respiratory rate per minute	32 (8)	40 (9)	27 (6)	42 (11)	39 (6)
Heart rate per minute	74 (12)	71 (22)	90 (13)	77 (12)	77 (12)
	**(Number calves, and % of total on each farm)**				
Dullness	2 (20)	0	0	0	0
Enopthalmus^c^	2 (20)	0	2 (20)	0	0
Eye discharge^d^	1 (10)	0	0	0	3 (38)
Nasal discharge^d^	5 (50)	2 (20)	2 (20)	3 (38)	6 (75)
Mouth ulcer	4 (40)	1 (10)	0	1 (13)	0
Muzzle ulcer	0	0	0	0	2 (25)
Hyper-salivation^e^	6 (60)	8 (80)	7 (70)	4 (50)	0
Diarrhoea	7 (78)^f^	7 (70)	8 (80)	2 (25)	7 (88)
Pneumonia	0	2 (20)	1 (10)	4 (50)	5 (63)
Poor coat	6 (60)	9 (90)	8 (80)	6 (75)	4 (50)

Age, body weight, rectal body temperature, respiratory rate, and heart rate are presented as mean (standard deviation) per farm; ^a^ The age of all calves in the group ranged from 118 to 149 days old on CF, while the age range of all calves in the group was not available for NCF; ^b^ Body weight at sampling; ^c^ Dull, sunken, or deeply sunken eyes; ^d^ Based on the presence or absence of discharge; ^e^ Deemed present during oral examinations; ^f^ Faecal sample not collected from one calf due to rectal emptying (7 out of 9 calves)

### Rumen fluid analysis

The mean ruminal pH of calves on CF and NCF were within normal reference levels (Table 6). Mean ammonia concentrations varied across farms, with the concentrations on CF 3 and NCF 2 below the minimum concentrations to support microbial growth. Mean concentrations of D-lactic acid, L-lactic acid, and DL-lactic acid were comparable between CF and NCF. Mean total VFAs concentration was lowest in rumen fluid samples from CF 1. The molar proportions of VFAs varied between farms, with acetate being the most abundant component. The acetate to propionate ratio was lowest on NCF 2.

**Table 6:** Mean (standard deviation) rumen fluid pH, ammonia, lactic acid, and volatile fatty acids (VFAs) concentrations on CF and NCF

Analyses	**CF**			NCF		Normal references
	**CF 1 (*n*** **= 10)**	**CF 2 (*n*** **= 10)**	CF 3 (*n* = 9)^+^	NCF 1 (*n* = 8)	NCF 2 (*n* = 6)^+^	
pH	7.09 (0.23)	6.75 (0.40)	6.67 (0.40)	6.88 (0.14)	6.43 (0.31)	> 5.8^a^
Ammonia (mg/L)	29.6 (15.6)	20.7 (18.5)	17.6 (15.8)	45.0 (8.8)	17.2 (8.8)	20^b^
D-Lactic acid (g/L)	0.07 (0.02)	0.06 (0.01)	0.08 (0.01)	0.08 (0.01)	0.06 (0.02)	< 0.09^c^
L-Lactic acid (g/L)	0.04 (0.01)	0.03 (0.01)	0.05 (0.01)	0.04 (0.01)	0.03 (0.01)	< 0.09^c^
DL-Lactic acid (g/L)	0.11 (0.03)	0.09 (0.02)	0.13 (0.01)	0.12 (0.02)	0.09 (0.30)	–
Total VFAs (m*M*)	153.6 (37.3)	243.2 (83.1)	286.0 (67.4)	273.5 (48.8)	250.9 (93.2)	–
Molar proportions of VFAs (mol/100 mol)						
Acetate	72.7 (3.0)	61.2 (3.9)	71.8 (2.8)	72.8 (2.0)	66.1 (1.1)	–
Propionate	16.7 (2.5)	20.2 (2.0)	16.5 (1.8)	14.9 (2.1)	22.6 (0.7)	–
Butyrate	7.0 (1.3)	13.6 (1.7)	8.8 (1.5)	10.0 (2.0)	8.9 (0.9)	–
Isobutyrate	1.1 (0.7)	0.8 (0.1)	0.4 (0.2)	0.6 (0.1)	0.5 (0.1)	–
Valeric	0.7 (0.2)	1.9 (0.9)	0.8 (0.6)	0.5 (0.1)	1.0 (0.3)	–
Isovaleric	1.8 (1.1)	2.3 (0.4)	1.7 (0.3)	1.2 (0.3)	0.9 (0.1)	–
Acetate:Propionate	4.5 (0.9)	3.1 (0.5)	4.4 (0.6)	5.0 (0.8)	2.9 (0.1)	–

^+^Calves with available rumen fluid samples on CF 3 (9 out of 10 calves) and NCF 2 (6 out of 8 calves); ^a^ Values for grazing dairy cows in Ireland [16]; ^b^ Minimum concentration to support microbial growth, with optimum microbial growth at 50 mg/L [17]; ^c^ Values for clinically healthy calves – adapted from Gentile et al. [18]

### Blood analyses

**Fig. 1:**
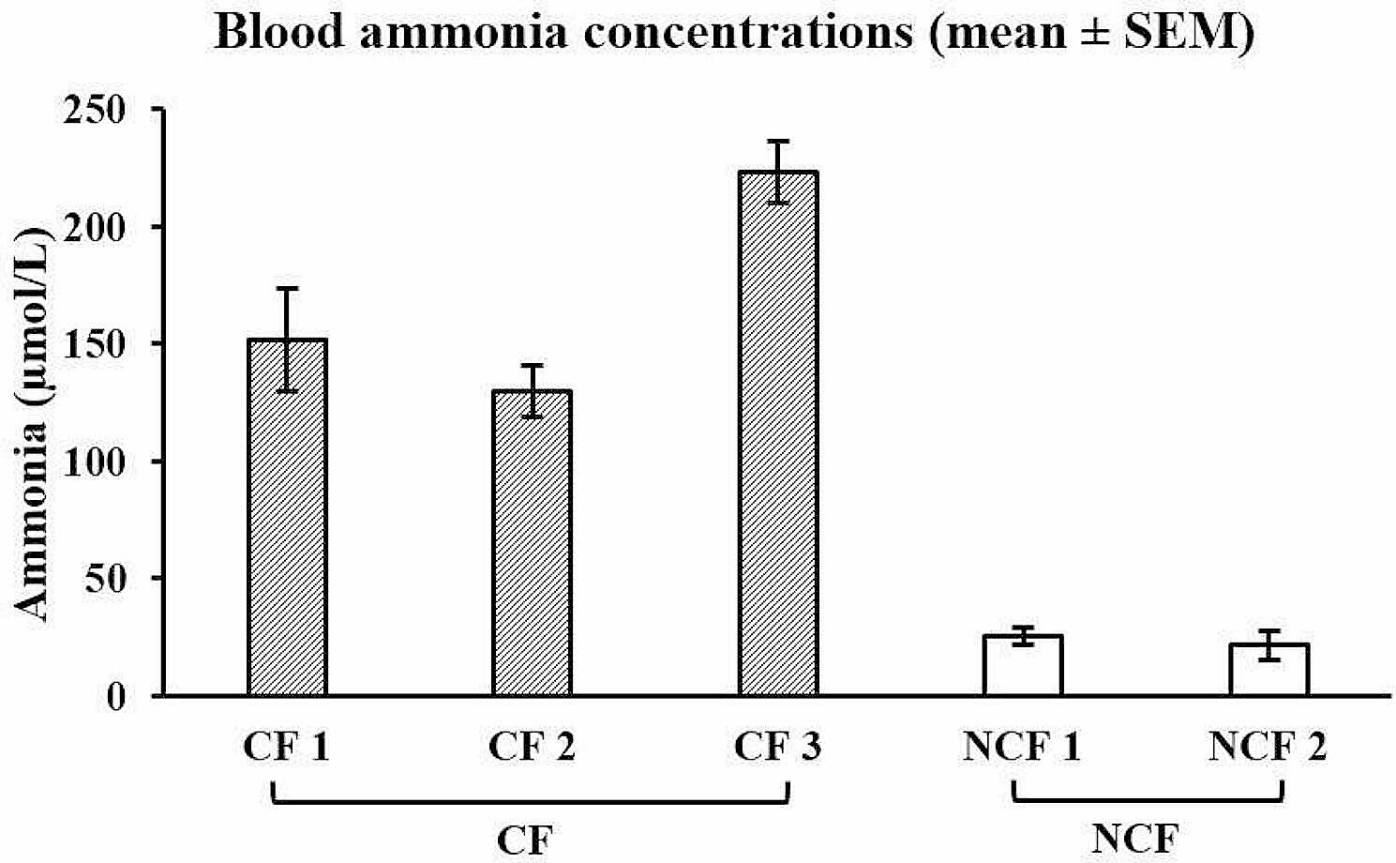
Mean blood ammonia concentrations on CF (129–223 µmol/L) and NCF (22–25 µmol/L) (normal < 90 µmol/L; [22])

The complete blood count, blood biochemistry, and blood mineral profiles of calves sampled on CF and NCF are summarised in Table 7. The majority of haematological variables had mean values within the normal range and were similar between CF and NCF. Calves originating from CF 1 and NCF 1 had mean CK activities that were above the normal range. On CF 1, CF 2, and NCF 1, the mean concentrations of GLDH were elevated from the normal range. The mean blood ammonia concentrations of all CF sampled calves exceeded the normal range and concentrations were 5 to 10 times greater than concentrations measured in NCF calves (Fig. 1). The mean concentrations of BUN of calves on CF 1 and CF 2 were similar and almost double the mean concentrations on CF 3 and NCF, but all were within the normal range. Calf blood cobalt concentrations exceeded the normal range on all farms, with the exception of CF 1. Mean blood molybdenum concentrations on NCF were double the concentrations on CF but all values were within the normal range. Similarly, the mean concentrations of the remaining blood minerals in calves from both CF and NCF were within the normal range.

**Table 7:** Complete blood count, blood biochemistry, and mineral profiles of calves on CF and NCF

Analyses	CF			NCF		Normal references^a, b^
	CF 1 (*n* = 10)	CF 2 (*n* = 10)	CF 3 (*n* = 10)	NCF 1 (*n* = 8)	NCF 2 (*n* = 8)	
***Complete blood count***						
WBC (×10^3^ cells/µL)	10.9 (2.2)	11.1 (2.5)	11.0 (2.1)	11.8 (3.2)	11.4 (4.0)	4.8–16.3
RBC (×10^6^ cells/µL)	10.4 (0.6)	9.6 (0.8)	9.9 (0.9)	9.8 (0.6)	10.5 (0.9)	6.2–11.9
Haemoglobin (g/dL)	12.9 (0.8)	11.8 (0.8)	13.1 (1.6)	12.1 (0.7)	12.6 (0.4)	7.3–14.8
Haematocrit (%)	30.5 (1.8)	29.0 (2.2)	30.8 (2.4)	30.1 (1.3)	30.9 (2.3)	23–45
Platelet (×10^3^ cells/µL)	470 (74)	623 (112)	560 (107)	674 (194)	671 (272)	238–1213
Neutrophil (×10^3^ cells/µL)	3.0 (1.8)	3.1 (1.1)	3.5 (1.1)	4.1 (2.5)	3.4 (1.8)	0.9–13.0
Lymphocyte (×10^3^ cells/µL)	5.6 (1.0)	6.0 (1.4)	6.8 (1.2)	6.9 (1.6)	7.1 (2.6)	0.1–8.4
Monocyte (×10^3^ cells/µL)	1.9 (0.7)	1.5 (0.5)	0.4 (0.2)	0.5 (0.1)	0.6 (0.3)	0–1.7
Eosinophil (×10^3^ cells/µL)	0.11 (0.09)	0.04 (0.01)	0.12 (0.05)	0.13 (0.11)	0.15 (0.06)	0–0.4
Basophil (×10^3^ cells/µL)	0.10 (0.04)	0.12 (0.07)	0.15 (0.03)	0.15 (0.05)	0.20 (0.08)	0–0.3
***Blood biochemistry***						
Albumin (g/L)	40.4 (1.4)	37.5 (3.0)	40.3 (3.0)	37.9 (2.4)	35.7 (2.8)	27–36
Total protein (g/L)	71.3 (2.8)	69.1 (3.0)	71.8 (4.0)	73.9 (5.5)	70.5 (4.8)	45–82
Globulin (g/L)	31.0 (2.5)	31.6 (1.7)	31.4 (3.9)	36.0 (6.3)	34.8 (5.1)	14–53
CK (µ/L)	373 (73)	271 (47)	275 (74)	363 (119)	311 (193)	46–326
GGT (U/L)	23.7 (7.4)	22.7 (5.4)	16.2 (5.7)	19.3 (5.7)	24.8 (2.7)	11.0–27.6^c^
GLDH (U/L)	93 (60)	95 (69)	19 (11)	94 (76)	53 (63)	5–60
Ammonia (µmol/L)	152 (49)^+^	129 (35)	223 (42)	25 (11)	22 (18)	< 90^d^
BUN (mmol/L)	6.8 (4.6)	6.6 (4.4)	3.7 (1.9)	3.2 (1.3)	3.5 (1.0)	1.7–7.7
Ammonia: BUN	20:1^^^	20:1	60:1	8:1	6:1	9:1^e^
***Blood mineral profiles***						
Calcium (mmol/L)	2.5 (0.2)	2.5 (0.1)	2.5 (0.1)	2.5 (0.1)	2.5 (0.2)	2.4–3.1^f^
Cobalt (µg/L)	1.7 (0.7)	3.1 (3.3)	2.6 (0.9)	3.5 (4.9)	12.0 (5.0)	0.17–2.00
Copper (µmol/L)	14.4 (1.7)	14.8 (1.3)	15.4 (1.4)	15.7 (4.0)	14.4 (1.4)	9.4–17.3
Magnesium (mmol/L)	1.01 (0.12)	0.99 (0.11)	1.00 (0.10)	1.00 (0.12)	1.00 (0.08)	0.74–1.10^f^
Molybdenum (µg/L)	6.5 (3.5)	6.6 (2.4)	7.5 (2.6)	13.3 (5.1)	12.4 (8.6)	2–35
Phosphorus (mmol/L)	2.1 (0.3)	2.1 (0.2)	2.0 (0.3)	2.0 (0.3)	2.2 (0.5)	1.8–2.1^f^
Selenium (µg/L)	68.9 (7.7)	66.1 (12.2)	78.5 (8.5)	63.3 (11.4)	80.7 (14.5)	65–140
Zinc (umol/L)	19.7 (2.1)	18.3 (2.7)	18.7 (3.0)	16.3 (1.7)	17.0 (4.5)	9.2–29.0

All results are presented as mean (standard deviation) per farm; WBC – white blood cells; RBC – red blood cells; CK – creatinine kinase; BUN – blood urea nitrogen; GGT – gamma glutamyl transferase; GLDH – glutamate dehydrogenase; ^+^ Blood ammonia results were only available for five calves; ^^^ Ammonia: BUN of five calves with complete blood ammonia results; ^a^ Reference for blood biochemistry and complete blood count – adopted from Roadknight et al. [19]; ^b^ Reference for blood mineral profiles – adopted from Herdt and Hoff [20]; ^c^ Yu et al. [21]; ^d^ Buczinski et al. [22]; ^e^ West [23]; ^f^ Constable et al. [24]

## Discussion

### General SSS case presentation

The onset of SSS clinical signs in the present study was reported between 2 and 6 weeks after the calves were turned out to grass. This is consistent with the observations that SSS commonly occurs in calves within a month after turnout to grass [2, 3]. The time gap between turnout and the onset of SSS clinical signs in affected calves is interesting. It may suggest that the syndrome is not an acute condition that occurs suddenly at grass but slowly progresses until it reaches a certain critical threshold of severity before clinical signs are observed, potentially related to the variation in grass growth and quality. Calves with SSS predominantly had diarrhoea, weight loss, poor coat condition, and a subset of calves had what appeared to be hyper-salivation. Although these clinical presentations are commonly observed in calves with SSS [3], however, they are not pathognomonic. Hunnam et al. [4] contended that the presence of clinical signs in affected calves within a herd is not sufficient to distinguish herds with or without UAUS and that post-mortem and histopathological examinations are required to confirm a diagnosis. However, using the aforementioned diagnostic approach requires post-mortem examination of a dead calf or of an affected calf that had been euthanized, and this was beyond the scope of the present study. The approach to SSS diagnosis in the present study was based on collecting farm and clinical data as part of the herd investigation, and eliminating the potential plausible differential diagnoses, which is consistent with the current diagnostic approach that is being applied in Ireland and the UK [3]. There is no consensus on either an animal-level or farm-level case definition for this syndrome [3, 4]. Nonetheless, the present study does suggest that the clinical presentation of this syndrome is broadly similar across farms in Ireland, with affected calves exhibiting diarrhoea and loss of condition in their first grazing season, which cannot be explained by common plausible causes such as coccidiosis or PGE.

### Hyperammonemia in SSS calves

An important finding in the present study was the high blood ammonia concentrations (hyperammonemia) in affected calves on CF. A study by Gultekin et al. [10] reported that calves with acute diarrhoea, irrespective of the aetiology, had elevated blood ammonia concentrations that were double the concentrations found in healthy calves, as measured using the point-of-care blood ammonia analyser. Interestingly, although the median blood ammonia concentrations were within the normal range, some calves with infectious diarrhoea had blood ammonia concentrations that exceeded the normal range (normal < 90 µmol/L; [22]), which is similar to the blood ammonia concentrations found in SSS calves in the present study. This similarity could suggest potential infectious aetiology for diarrhoea and hyperammonemia in SSS calves. However, it is important to note that the infectious agents (e.g., C*ryptosporidium parvum, Escherichia coli*, rotavirus, and coronavirus) identified in that study commonly cause diarrhoea in calves less than a month old [25], which was not the case for calves (age more than 3 months old) in the present study. Additionally, the clinical presentations, epidemiological pattern, and historic case presentations of SSS calves are not consistent with infectious aetiologies. The level of blood ammonia concentrations in the present study appears to have an inconsistent pattern in relation to the severity of diarrhoea (see Additional file 1), and is contrary to the findings of Gultekin et al. [10], who reported a positive relationship between blood ammonia concentrations and severity of diarrhoea. This discrepancy raises questions about the potential causal relationship between diarrhoea and hyperammonemia in SSS calves, specifically whether the presence of diarrhoea leads to hyperammonemia or vice versa. A recent surveillance report by Swinson et al. [3] suggested that the histopathological lesions in calves with SSS are indicative of potential chemical insults, such as those caused by acidosis and high blood urea or ammonia. However, the authors of that study did not measure blood ammonia concentrations in SSS calves. Nonetheless, due to the absence of histopathological examinations in the present study, the relationship between hyperammonemia and the formation of histopathological lesions in SSS calves is speculative and warrants further investigation.

### Potential cause for hyperammonemia in SSS calves

In the present study, farmers on CF reported recent application (1 to 3 weeks) of inorganic nitrogen fertilisers (i.e., protected urea and NPK) prior to grazing. Excessive application of inorganic nitrogen fertiliser can increase the amount of nitrogen in the soil that is taken up by the grass, resulting in higher herbage nitrogen and CP [14, 26]. Interestingly, the grass samples from the paddocks where the affected calves had grazed (137–148 g/kg DM), and were currently grazing on the day of visit (106–177 g/kg DM) had low to moderate CP concentrations. This finding was also surprising and may not agree with the hypothesis on the association between grazing highly fermentable grass and SSS. The grazing of lush grass that is high in CP (greater than 200 g/kg DM; [27]) and low in fibre (less than 400 g/kg DM; [27]) is a common factor that is often associated with SSS [1]. However, the results of grass analysis in the present study should be interpreted with caution, as the stage of grass growth at the time of sampling may not have been representative of the grass condition when the calves were turned out or when the onset of SSS clinical signs was first evident. Furthermore, the time of investigation and grass sampling (June 2023) was exceptionally dry, and lush grass growth expected at that time of the year was not available. Grass chemical composition varies depending on the stage of grass growth [28]. It is possible that the grass was lush at the time of turnout or at the onset of SSS clinical signs and had grown to stemmy grass at the time of farm visits and sample collections.

Upon ingestion by the calves, grass nitrogen is converted into ammonia by rumen bacteria, mediated by urease activities [29]. If there is an imbalance between the ammonia produced from nitrogen metabolism and the rate of ammonia that can be utilised for microbial protein synthesis, this condition leads to excess ammonia accumulation in the rumen, and the ammonia will then be absorbed into the bloodstream [29]. The ruminal ammonia concentrations in the present study were not indicative of excess accumulation (below the optimum concentration of 50 mg/L; [17]), and appear to be consistent with the grass CP concentrations [14, 26]. However, it was surprising that there was a mismatch in the increments of blood ammonia and ruminal ammonia concentrations. One possible explanation could be related to the disruption in the mechanism of ammonia clearance post-ruminal. Under normal physiological conditions, systemic ammonia is converted into urea in the liver before being further excreted by the kidney [29]. However, in cases of liver dysfunction, this conversion process is impaired, resulting in hyperammonemia [29]. This underlying mechanism may explain the simultaneous findings of hyperammonemia and high GLDH concentrations, an indicator of liver damage [30] in affected calves on CF 1 and CF 2, although the increments in GLDH concentrations may be insufficient to indicate severe liver damage. An increase in liver enzymes of more than 10-fold is generally indicative of severe liver damage, while an increase of less than 5-fold is considered mild to moderate [31]. Interestingly, the decreased BUN concentration that is typically associated with liver damage [32] was not observed in the present study. A study by West [23] reported that the blood ammonia: urea in normal healthy cattle was 9:1 while a ratio greater than 30:1 was an indicator of liver damage. Unlike plasma urea, which reflects degradable protein intake, plasma ammonia levels are more variable and dependent on the liver for removal. In the present study, 16 of the 25 calves on CF with complete blood ammonia results had a blood ammonia: urea greater than 30:1. This finding further supports the liver damage as potential mechanism for hyperammonemia. Cattle with liver disease are reported to have clinical manifestations such as depression, anorexia, weight loss, and diarrhoea [23]. Nonetheless, it is also possible that other factors or yet undetermined underlying mechanisms may contribute to the hyperammonemia in the present study, particularly in affected calves without indication of liver damage (blood ammonia: urea less than 30:1). Other factors, such as overgrowth of ammonia-producing microorganisms [33] and deficiency of urea cycle enzymes [34] have been suggested as causes of hyperammonemia without liver damage in other animal species.

There may also an indirect association between the timing and level of fertiliser application and hyperammonemia detected in SSS calves, specifically due to potential nitrate or non-protein nitrogen (NPN; e.g., urea) poisonings as a result of fertiliser use [35, 36]; but that is of course if the hyperammonemia findings are relevant to the development of the clinical symptoms such as diarrhoea. Clinical signs such as hyper-salivation and/or diarrhoea have been observed in ruminants with these types of poisonings [37, 38], which is consistent with the clinical signs observed in SSS calves in the present study. However, it is important to note that cases of nitrate or NPN poisonings are often acute and fatal, where animals usually exhibit neurological clinical signs or die within hours [35, 36]. No calf deaths were reported in the present study, suggesting that acute nitrate or NPN poisonings were unlikely causes of SSS. Nonetheless, it is also probable that SSS calves consumed grass that contained sub-lethal nitrate and NPN over time, causing chronic nitrate or NPN poisonings [39]. Chronic nitrate poisonings have been reported to cause reproductive problems in adult cattle [40]; however, to our knowledge no studies have reported effects on growing calves.

### Differential diagnoses of SSS

Several differential diagnoses of SSS were considered in the present study to inform the sample strategy during herd investigation, including coccidiosis, PGE, mineral deficiencies or toxicities (e.g., copper or molybdenum), and ruminal acidosis, as each of these conditions can cause calf diarrhoea at grass [25]. None of the above conditions were evident, as parasitology analysis of faecal samples from CF found a low parasitic burden, and blood copper and molybdenum concentrations were within the normal range. Moreover, the ruminal pH indicated normal rumen acidity, and the alteration of D-lactic acid concentrations in ruminal fluid– which is commonly reported in cases of ruminal acidosis [18], was not observed. However, the normal ruminal pH might be questionable due to the potential saliva contamination during sample collections and the farmers supplemented the affected calves with ruminate powders or buffers, which may have increased the ruminal pH [41, 42]. Nonetheless, affected calves continued to show clinical signs despite having normal ruminal pH may also suggest that rumen acidosis is unlikely to be the primary cause of SSS. All of the visited farms had a BVDV-negative status, thus ruling out BVDV as a possible cause of infection. Other differential diagnoses, such as *Salmonella* infections, were not investigated in the present study. In the case of *Salmonella* infection, diarrhoea is generally presented with mucus or traces of blood that are frequently accompanied by fever and neutropenia [43, 44] due to the systemic nature of the infection. These conditions were not observed in affected calves in the present study.

### Challenge the widespread hypothesis of SSS causes

Anecdotal reports in Ireland suggest that the cause of SSS could be related to inadequate rumen development. In conditions where the rumen is inadequately developed, there may be an imbalance in microbial composition, which can disrupt the digestive process in the rumen [45]. This can lead to an accumulation of undigested or unfermented grass substances in the small intestines, resulting in malabsorption and subsequently diarrhoea [46]. Another consequence is related to the reduction of rumen capacity to absorb VFAs, leading to higher accumulation of VFAs in the rumen and decreased rumen pH [47]. The total VFAs concentrations in the present study, either from calves sampled on CF or NCF, were numerically greater than the values reported by O’Grady et al. [16], in grazing Irish dairy cows. Although the high VFAs concentrations could indicate potential VFAs accumulation, it is important to note that the concentration of VFAs in rumen fluid generally increases after feeding, and its relationship with rumen pH is influenced by diet [47]. Calves in the present study had received concentrate supplementation before rumen fluid collection, which may explain the high VFAs concentrations. Intervention in early feeding management during the pre-weaning period is crucial in promoting rumen microbial colonization and subsequent rumen development [45, 48]. Studies have reported that the introduction of step-down milk feeding [49], concentrate feeding [50], and forage provision [51] during the pre-weaning period facilitates rumen development. Considering those effects of early feeding practices on rumen development, it is likely that the pre-weaning feeding practices (e.g., step-down milk feeding, concentrate feeding, and forage provision pre- and post-weaning) implemented on CF have stimulated adequate rumen development in calves. However, despite many studies and reviews published in recent years that evaluated different calves’ feeding strategies on performance and health [52–57], there is a concern that calves are commonly underfed or receive inadequate amounts of milk feeding, which results in suboptimal growth performance and organ development. While inadequate milk or milk replacer feeding practices and inadequate rumen development could be potential contributing factors for SSS occurrence, further research is warranted to investigate their involvement.

### Study limitations

The time lapse between the onset of SSS clinical signs and sample collections could be considered a limitation of the present study. Ideally, the biological samples or grass samples should be collected shortly after the onset of SSS clinical signs in order to accurately reflect the conditions that potentially trigger the SSS occurrence. However, this is a challenge in field studies as there is often a delay from the times that farmers observe the SSS clinical signs until the reporting of cases. For example, farmers on CF observed calves displaying SSS clinical signs between 2 days and 2.5 weeks before the farm visits and sample collections. Consequently, it is possible that the affected calves had already passed the ‘peak’ period of the SSS clinical signs and had started to recover, albeit they still exhibited the clinical signs at the time of the farm visits. The calves selection for clinical examination was somewhat ad hoc owing to the nature of clinical investigation, and in addition the farmers reported most of the calves in the group were affected but to varying severity. This lack of clarity and robust numbers of affected calves in the group is considered weakness of the present study. Another limitation could possibly be the sensitivity and specificity of the semi-quantitative point-of-care ammonia analyser used in the present study, which have not been determined in calves. However, a study in small animals provides supporting evidence that the identical point-of-care ammonia analyser (PocketChem BA) has adequate precision and positive linearity (*R*^*2*^ = 0.98), and negative proportional bias (*R*^*2*^ = 0.71) with the standard enzymatic method [11]. Lastly, the self-selected nature of farms referred for herd investigation and the limited number of farms reporting the cases in June 2023 may underestimate the true occurrence of SSS on Irish commercial farms.

## Conclusion

The present study provides interesting information on the occurrence and characteristics of SSS on Irish commercial farms. The findings suggest that copper or molybdenum toxicities, and ruminal acidosis are not the potential causes of SSS, and that ruling out common causes of diarrhoea at grass seems a logical approach to defining the SSS condition. In addition, the present study provides further evidence that calves with SSS present with clinical signs of diarrhoea and loss of condition after recent turnout to grass for first time grazing. The presence of hyperammonemia in calves with SSS and the potential association with the timing and level of inorganic nitrogen fertiliser application prior to calves grazing the paddocks warrant further investigation to determine the potential definitive cause of SSS.

### Electronic supplementary material

Below is the link to the electronic supplementary material.

Supplementary Material 1

## Data Availability

The data generated during this study will be made available upon reasonable request to Dr Bernadette Earley (bernadette.earley@teagasc.ie).

## Abbreviations

AA: Aberdeen angus
ADF: Acid detergent fibre
BA: Blonde d’Aquitane
BRD: Bovine respiratory disease
BUN: Blood urea nitrogen
BVDV: Bovine viral diarrhoea virus
CF: Case farms
CK: Creatinine kinase
CP: Crude protein
DM: Dry matter
DMD: Dry matter digestibility
DOMD: Dry organic matter digestibility
FR: Friesian
GGT: Gamma glutamyl transferase
GLDH: Glutamate dehydrogenase
HO: Holstein
ICBF: Irish Cattle Breeding Federation
LM: Limousin
NCF: Non-case farms
NDF: Neutral detergent fibre
NPK: Nitrogen, phosphorus, and potassium
NPN: Non-protein nitrogen
OAD: Once a day
OMD: Organic dry matter
PGE: Parasitic gastroenteritis
PVP: Private veterinary practitioners
RBC: Red blood cells
SSS: Summer scours syndrome
TAD: Twice a day
TCA: Trichloroacetic acid
UAUS: Upper alimentary ulcerative syndrome
UK: United Kingdom
VFAs: Volatile fatty acids
WBC: White blood cells
WSC: Water-soluble carbohydrate

## References

[1] RVL (2021). Regional veterinary laboratories report. Vet Irel J.

[2] Hateley G, Mason C, Henderson K, Fagan S, Millar M, Neale S (2018). Severe summer scour syndrome in recently turned out dairy calves. Vet Rec.

[3] Swinson V, Nabb L, Henderson K, Millar M (2023). Update on severe summer scour syndrome in cattle. Vet Rec.

[4] Hunnam JC, Jerrett IV, Mee PT, Moore K, Lynch SE, Rawlin GT (2021). An idiopathic upper alimentary tract ulcerative syndrome in weaned dairy calves in Victoria, Australia. Transbound Emerg Dis.

[5] Cuevas-Gómez I, McGee M, Sánchez JM, O’Riordan E, Byrne N, McDaneld T (2021). Association between clinical respiratory signs, lung lesions detected by thoracic ultrasonography and growth performance in pre-weaned dairy calves. Ir Vet J.

[6] Donlon JD, Mee JF, McAloon CG. Prevalence of respiratory disease in Irish preweaned dairy calves using hierarchical bayesian latent class analysis. Front Vet Sci. 2023;10(4). 10.3389/fvets.2023.1149929 PMC10133517 37124570

[7] Renaud DL, Buss L, Wilms JN, Steele MA (2020). Technical note: is fecal consistency scoring an accurate measure of fecal dry matter in dairy calves?. J Dairy Sci.

[8] O’Shaughnessy J, Earley B, Mee JF, Doherty ML, Crosson P, Barrett D (2015). Controlling nematodes in dairy calves using targeted selective treatments. Vet Parasitol.

[9] Joachim A, Altreuther G, Bangoura B, Charles S, Daugschies A, Hinney B (2018). W A A V P guideline for evaluating the efficacy of anticoccidials in mammals (pigs, dogs, cattle, sheep). Vet Parasitol.

[10] Gultekin M, Voyvoda H, Ural K, Erdogan H, Balikci C, Gultekin G (2019). Plasma citrulline, arginine, nitric oxide, and blood ammonia levels in neonatal calves with acute diarrhea. J Vet Intern Med.

[11] Goggs R, Serrano S, Szladovits B, Keir I, Ong R, Hughes D (2008). Clinical investigation of a point-of-care blood ammonia analyzer. Vet Clin Pathol.

[12] McGettigan CE, McGee M, O’Riordan EG, Kelly AK, Earley B (2022). Effect of concrete slats versus rubber-covered slats on the performance, behaviour, hoof health, cleanliness of finishing beef steers and performance, cleanliness and hoof health of weanling cattle. Livest Sci.

[13] Smith PE, Waters SM, Kenny DA, Kirwan SF, Conroy S, Kelly AK (2021). Effect of divergence in residual methane emissions on feed intake and efficiency, growth and carcass performance, and indices of rumen fermentation and methane emissions in finishing beef cattle. J Anim Sci.

[14] O’Connor A, Moloney A, O’Kiely P, Boland T, McGee M (2019). Effects of fertiliser nitrogen rate to spring grass on apparent digestibility, nitrogen balance, ruminal fermentation and microbial nitrogen production in beef cattle and in vitro rumen fermentation and methane output. Anim Feed Sci Technol.

[15] O’Kiely P (2011). Intake, growth and feed conversion efficiency of finishing beef cattle offered diets based on triticale, maize or grass silages, or ad libitum concentrate. Ir J Agric Food Res.

[16] O’Grady L, Doherty ML, Mulligan FJ (2008). Subacute ruminal acidosis (SARA) in grazing Irish dairy cows. Vet J.

[17] Satter LD, Slyter LL (1974). Effect of ammonia concentration on rumen microbial protein production in vitro. Br J Nutr.

[18] Gentile A, Sconza S, Lorenz I, Otranto G, Rademacher G, Famigli-Bergamini P (2004). D-Lactic acidosis in calves as a consequence of experimentally induced ruminai acidosis. J Vet Med Ser Physiol Pathol Clin Med.

[19] Roadknight NW, Courtman NF, Mansell PD, Jongman EC, Loh ZA, Fisher AD (2021). Biochemistry and hematology reference intervals for neonatal dairy calves aged 5–12 days. Vet Clin Pathol.

[20] Herdt TH, Hoff B (2011). The use of blood analysis to evaluate trace mineral status in ruminant livestock. Vet Clin North Am - Food Anim Pract.

[21] Yu K, Canalias F, Solà-Oriol D, Arroyo L, Pato R, Saco Y (2019). Age-related serum biochemical reference intervals established for unweaned calves and piglets in the post-weaning period. Front Vet Sci.

[22] Buczinski S, Duval J, d’Anjou MA, Francoz D, Fecteau G (2007). Portacaval shunt in a calf: clinical, pathologic, and ultrasonographic findings. Can Vet J.

[23] West HJ (1997). Clinical and pathological studies in cattle with hepatic disease. Vet Res Commun.

[24] Constable PD, Hinchcliff KW, Done SH, Grünberg W (2017). Reference laboratory values. Veterinary Medicine.

[25] Blanchard PC (2012). Diagnostics of dairy and beef cattle diarrhea. Vet Clin North Am Food Anim Pract.

[26] O’Connor A, McGee M, Moloney A, Boland T, O’Kiely P (2019). Digestion and nitrogen metabolism in beef cattle and in vitro rumen fermentation of autumn grass differing in fertilizer nitrogen application rate. Grass Forage Sci.

[27] Bargo F, Varga GA, Muller LD, Kolver ES (2003). Pasture intake and substitution rate effects on nutrient digestion and nitrogen metabolism during continuous culture fermentation. J Dairy Sci.

[28] King C, McEniry J, Richardson M, O’Kiely P (2012). Yield and chemical composition of five common grassland species in response to nitrogen fertiliser application and phenological growth stage. Acta Agric Scand Sect B Soil Plant Sci.

[29] Patra AK, Aschenbach JR (2018). Ureases in the gastrointestinal tracts of ruminant and monogastric animals and their implication in urea-N/ammonia metabolism: a review. J Adv Res.

[30] Hoffmann WE, Solter PF, Kaneko J, Harvey JW, Bruss ML (2008). Diagnostic enzymology of domestic animals. Clinical biochemistry of domestic animals.

[31] Webster CR, Cooper J, Bonagura J, Twedt D (2014). Diagnostic approach to hepatobiliary disease. Kirk’s current Veterinary Therapy XV.

[32] Johnson SE, Sherding RG, Birchard SJ, Sherding RG (2006). Diseases of the liver and biliary tract. Saunders Manual of Small Animal Practice.

[33] Stickle JE, McKnight CA, Williams KJ, Carr EA (2006). Diarrhea and hyperammonemia in a horse with progressive neurologic signs. Vet Clin Pathol.

[34] Zandvliet MMJM, Rothuizen J (2007). Transient hyperammonemia due to urea cycle enzyme deficiency in Irish wolfhounds. J Vet Intern Med.

[35] Radke S. Toxicology basics: Common toxicants in bovine medicine. AABP Proc. 2021;54(2).

[36] Cope RB, Gupta RC (2018). Nonprotein nitrogen (urea) and hyperammonemia. Veterinary toxicology.

[37] Binta M, Mushi E (2012). Environmental factors associated with nitrate poisoning in livestock in Botswana. J Pet Environ Biotechnol.

[38] Whitehair CK. Urea (ammonia) toxicosis in cattle. Bov Pract. 1989;67–73.

[39] Molín J, Mendonça FS, Henderson EE, Nyaoke AC, Ramírez GA, Navarro MA (2021). Toxic wasting disorders in sheep. Animals.

[40] Ozmen O, Mor F, Sahinduran S, Unsal A (2005). Pathological and toxicological investigations of chronic nitrate poisoning in cattle. Toxicol Environ Chem.

[41] Jaramillo-López E, Itza-Ortiz MF, Peraza-Mercado G, Carrera-Chávez JM (2017). Ruminal acidosis: strategies for its control. Austral J Vet Sci.

[42] Duffield T, Plaizier JC, Fairfield A, Bagg R, Vessie G, Dick P (2004). Comparison of techniques for measurement of rumen pH in lactating dairy cows. J Dairy Sci.

[43] Van Metre D, Divers TJ, Peek SF (2008). Infectious diseases of the gastrointestinal tract. Rebhun’s diseases of dairy cattle.

[44] Mohler VL, Izzo MM, House JK (2009). Salmonella in calves. Vet Clin NA Food Anim Pract.

[45] Du Y, Gao Y, Hu M, Hou J, Yang L, Wang X et al. Colonization and development of the gut microbiome in calves. J Anim Sci Biotechnol. 2023;1–15. 10.1186/s40104-023-00856-x PMC10082981 37031166

[46] Naylor JM, Anderson DE, Rings DM (2008). Neonatal calf diarrhea. Food Animal Practice.

[47] Dijkstra J, Ellis JL, Kebreab E, Strathe AB, López S, France J (2012). Ruminal pH regulation and nutritional consequences of low pH. Anim Feed Sci Technol.

[48] Diao Q, Zhang R, Fu T (2019). Review of strategies to promote rumen development in calves. Animals.

[49] Khan MA, Lee HJ, Lee WS, Kim HS, Ki KS, Hur TY (2007). Structural growth, rumen development, and metabolic and immune responses of Holstein male calves fed milk through step-down and conventional methods. J Dairy Sci.

[50] Suárez BJ, Van Reenen CG, Gerrits WJJ, Stockhofe N, Van Vuuren AM, Dijkstra J (2006). Effects of supplementing concentrates differing in carbohydrate composition in veal calf diets: II. Rumen development. J Dairy Sci.

[51] Castells L, Bach A, Aris A, Terré M (2013). Effects of forage provision to young calves on rumen fermentation and development of the gastrointestinal tract. J Dairy Sci.

[52] Khan MA, Bach A, Weary DM, von Keyserlingk MAG. Invited review: Transitioning from milk to solid feed in dairy heifers. J Dairy Sci. 2016;99(2):885–902. 10.3168/jds.2015-9975 26709160

[53] Kertz AF, Hill TM, Quigley JD, Heinrichs AJ, Linn JG, Drackley JK. A 100-year review: Calf nutrition and management. J Dairy Sci. 2017;100(12):10151–72. 10.3168/jds.2017-13062 29153160

[54] Johnson KF, Chancellor N, Burn CC, Wathes DC. Analysis of pre-weaning feeding policies and other risk factors influencing growth rates in calves on 11 commercial dairy farms. Animal. 2018;12(7):1413–23. 10.1017/S1751731117003160 29166977

[55] Hammon HM, Liermann W, Frieten D, Koch C. Review: Importance of colostrum supply and milk feeding intensity on gastrointestinal and systemic development in calves. Animal. 2020;14(S1):S133–43. 10.1017/S1751731119003148 32024575

[56] Lorenz I. Calf health from birth to weaning – an update. Ir Vet J. 2021;74(1):5. 10.1186/s13620-021-00185-3 PMC7968278 33726860

[57] Ockenden EM, Russo VM, Leury BJ, Giri K, Wales WJ. Preweaning nutrition and its effects on the growth, immune competence and metabolic characteristics of the dairy calf. Animals. 2023;13(5). 10.3390/ani13050829 PMC10000027 36899685

