# Supplementary material for: Summer scour syndrome in weaned dairy calves: case series
Source: Ir Vet J. 2024 Jul 16;77:14. doi: 10.1186/s13620-024-00273-0 (PMC11251238; doi:10.1186/s13620-024-00273-0)
Supplement: Supplementary file 1 — Supplementary Material 1 [file 13620_2024_273_MOESM1_ESM.pdf]

Additional file 1.

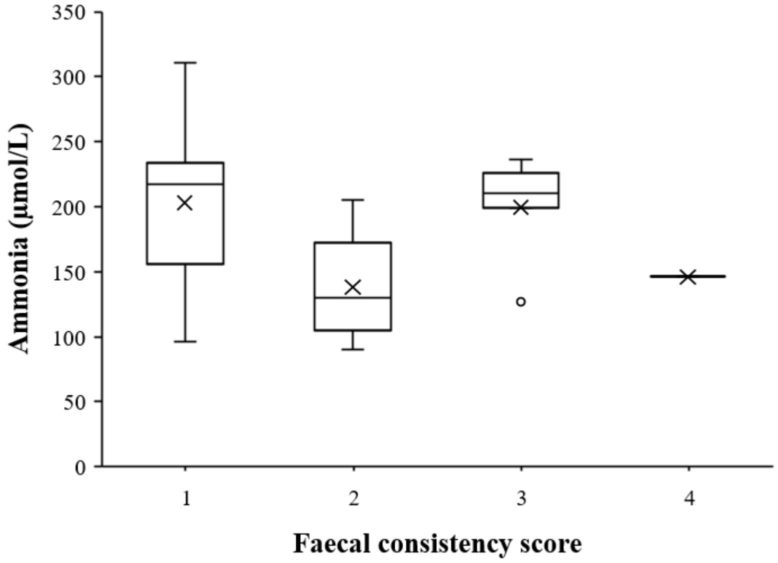

Additional file 1. Boxplot representing blood ammonia concentrations and severity of diarrhoea based on the faecal consistency score in SSS calves sampled on CF (n = 25). Faecal consistency score: 1) runny, liquid consistency, splatters on impact, spreads rapidly; 2) loose (watery), may pile slightly, spreads and splatters moderately on impact and settling; 3) soft, piles but spreads slightly on impact and settling; 4) dry, hard, dry appearance, original form not distorted on impact and settling.
